# Using a Wearable-Based Animated Patient Avatar to Improve Patients’ Perception of Vital Signs: Multicenter Computer-Based Study

**DOI:** 10.2196/84130

**Published:** 2026-03-12

**Authors:** Johannes Köhler, Max Ebensperger, Cynthia A Hunn, Arend Rahrisch, Achilles Delis, Florian Piekarski, Gregor Massoth, Florian J Raimann, Kai Zacharowski, David W Tscholl, Tadzio Raoul Roche

**Affiliations:** 1 Institute for Anesthesiology and Perioperative Medicine University and University Hospital Zurich Zurich Switzerland; 2 Department of Anesthesiology and Intensive Care TUM School of Medicine and Health Technical University of Munich Munich Germany; 3 Department of Anesthesiology and Intensive Care Medicine University Hospital Bonn Bonn Germany; 4 Goethe University Frankfurt, University Hospital, Department of Anaesthesiology, Intensive Care Medicine and Pain Therapy Frankfurt Germany

**Keywords:** diagnosis, patient avatars, patient monitoring, situation awareness, vital sign information, usability, wearable

## Abstract

**Background:**

Visual patient avatars are an innovative patient monitoring technology that can be used to translate numerical and waveform data into intuitive, avatar-based representations of patient conditions. Previous research indicates that this technology improves health care providers’ situational awareness compared to conventional monitoring methods. As patient-worn continuous vital sign monitoring continues to evolve, we introduce the Visual Patient Wearable device to provide avatar-based visualization tailored to this application.

**Objective:**

This study aimed to evaluate whether a wearable-based animated patient avatar can improve patients’ perception and recall of simulated vital sign deviations compared to conventional monitoring methods, and to assess the usability and acceptance of this avatar-based visualization.

**Methods:**

This computer-based study included 67 patients from 3 academic hospitals in Central Europe. Participants were randomly assigned to a Visual Patient Wearable group or a conventional monitoring group and viewed a standardized instructional video for their allocated method. They then completed 4 randomized clinical scenarios, each displayed for 6 seconds to simulate glance-based assessment. Accuracy in recalling vital sign deviations was measured, and Visual Patient Wearable participants additionally gave user feedback on Likert scales.

**Results:**

The Visual Patient Wearable system was associated with higher detection accuracy of vital sign deviations compared with standard monitoring layouts, increasing from a median correctness of 46% (IQR 33%-63%) with conventional monitoring to a median of 67% (IQR 49%-79%) with the Visual Patient Wearable system (*P*<.001). This corresponded to a risk ratio of 1.34 (95% CI 1.23%-1.47%). The magnitude of this association varied across signals, with the largest relative improvement observed for heart rhythm (137% improvement, 95% CI 85%-209%), followed by oxygen saturation (SpO_2_; 64% improvement, 95% CI 30%-108%) and temperature (30% improvement, 95% CI 5%-60%). No statistically significant reductions in risk were observed for heart rate, respiratory rate, or blood pressure. User experience ratings based on Likert scale assessments indicated high levels of satisfaction across all 6 vital sign categories (median score 4, IQR 4-5 on a 5-point scale).

**Conclusions:**

This computer-based study suggests that Visual Patient Wearable visualizations enhance patients’ ability to detect and recall simulated vital sign deviations. Participants found the system intuitive, easy to learn, and reassuring. The Visual Patient Wearable system provides an at-a-glance interface that may support patients’ understanding of their vital signs and could facilitate communication of relevant information to clinical staff, thereby potentially contributing to informed patient engagement. The next step is to develop a software prototype for wearable devices and test it in a clinical study.

## Introduction

### Background

Visual patient avatars were first introduced in 2018 as a novel approach to presenting vital sign data by transforming traditional numerical and waveform information into an animated, avatar-based display [1]. This intuitive design improves the comprehensibility of vital signs, making physiological changes easier to interpret at a glance [2]. The development of visual patient avatars was grounded in the principles of situational awareness [3], which is recognized as critical for safe and effective decision-making in anesthesia and critical care settings [4,5]. Studies have demonstrated that avatar-based visualization enables clinicians to detect vital sign deviations more quickly and with greater confidence than standard displays, while also reducing cognitive workload [6,7]. Further investigations have demonstrated the robustness of the avatar concept across various clinical scenarios, including (simulated) high-stress environments [8,9], peripheral viewing conditions [10], and monitoring at a distance [11]. Visual patient avatars have since been applied successfully in operating rooms and acute care settings, including intensive care [12,13] and pediatric anesthesia [14].

Despite these advances in intraoperative and critical care monitoring, there remains a significant gap in continuous vital sign awareness on general hospital wards, where patients are typically monitored only intermittently [15]. Many adverse events, such as hypotensive episodes or desaturation events, occur in the perioperative setting and remain undetected for multiple hours [16,17]. The European Surgical Outcomes Study reported that 73% of patients who died after surgery were never admitted to critical care at any point [18]. Similarly, national audits from the United Kingdom indicate that more than half of in-hospital cardiac arrests occur on regular wards, often without prior warning [19].

Emerging technologies such as wireless wearable sensors promise continuous, real-time surveillance of vital signs on the ward [20]. These systems have been associated with reductions in desaturation episodes, intensive care unit transfers, and postoperative complications when compared to traditional intermittent monitoring [21]. However, current solutions predominantly rely on passive data collection and alarm-based alerts, offering limited patient involvement [22].

To address this gap, we introduce Visual Patient Wearable device, a patient-worn, avatar-based system that translates vital sign data into an intuitive format. Unlike conventional monitors, the Visual Patient Wearable system is designed for direct patient use, promoting engagement and supporting the concept of patient-centered precision care [23,24]. Conceptually, the Visual Patient Wearable system targets the first link in an intervention-outcome chain. By enabling patients to detect vital sign deviations early, it may support timely actions by patients or notified clinicians, potentially improving process measures such as response times and, over time, may contribute to a reduction in adverse outcomes such as desaturation episodes, intensive care unit transfers, and postoperative complications.

### Objectives

This study aimed to investigate whether an animated patient avatar displayed on a wearable device (the Visual Patient Wearable system) can enhance patients’ perception of their vital signs. Specifically, we aimed to determine (1) whether avatar-based visualization of vital sign data enhances patients’ ability to detect early deviations and (2) how patients perceive the learnability and utility of the Visual Patient Wearable system.

## Methods

### Ethical Considerations

The study received a declaration of nonjurisdiction (exemption from ethics approval) from the Cantonal Ethics Commission of Zurich, Switzerland (Req-2024-00286), as no personal health data were collected. In addition, the study obtained positive ethics approval from both participating sites in Germany (Bonn: 2024-209-BO; Frankfurt: 2024-1759). The study was conducted in accordance with the principles of the Declaration of Helsinki. All participants provided written informed consent before participation and were free to withdraw at any time without consequences. Nonparticipation or withdrawal did not affect patients or their subsequent medical treatment. No personal or identifiable data were collected. Participants were tested experimentally using arbitrary vital parameters. Individual medical parameters, medical history, diagnoses, or therapies were neither collected, manipulated, nor stored. All data were fully anonymized at the time of collection and stored securely on password-protected systems accessible only to the study researchers. Participants did not receive any financial or other compensation for participation. Reporting followed the Guidelines for Health Care Simulation Research and extensions to the CONSORT (Consolidated Standards of Reporting Trials) and STROBE (Strengthening the Reporting of Observational Studies in Epidemiology) statements [25].

### Study Design

This computer-based study compared patients’ ability to correctly identify simulated vital sign deviations (respiratory rate, oxygen saturation [SpO_2_], temperature, blood pressure, heart rate, and heart rhythm) presented on a wearable device using two visualization methods: (1) a conventional numeric display with 1-lead electrocardiogram and (2) an animated patient-avatar display based on existing visual patient avatars.

**Figure 1:**
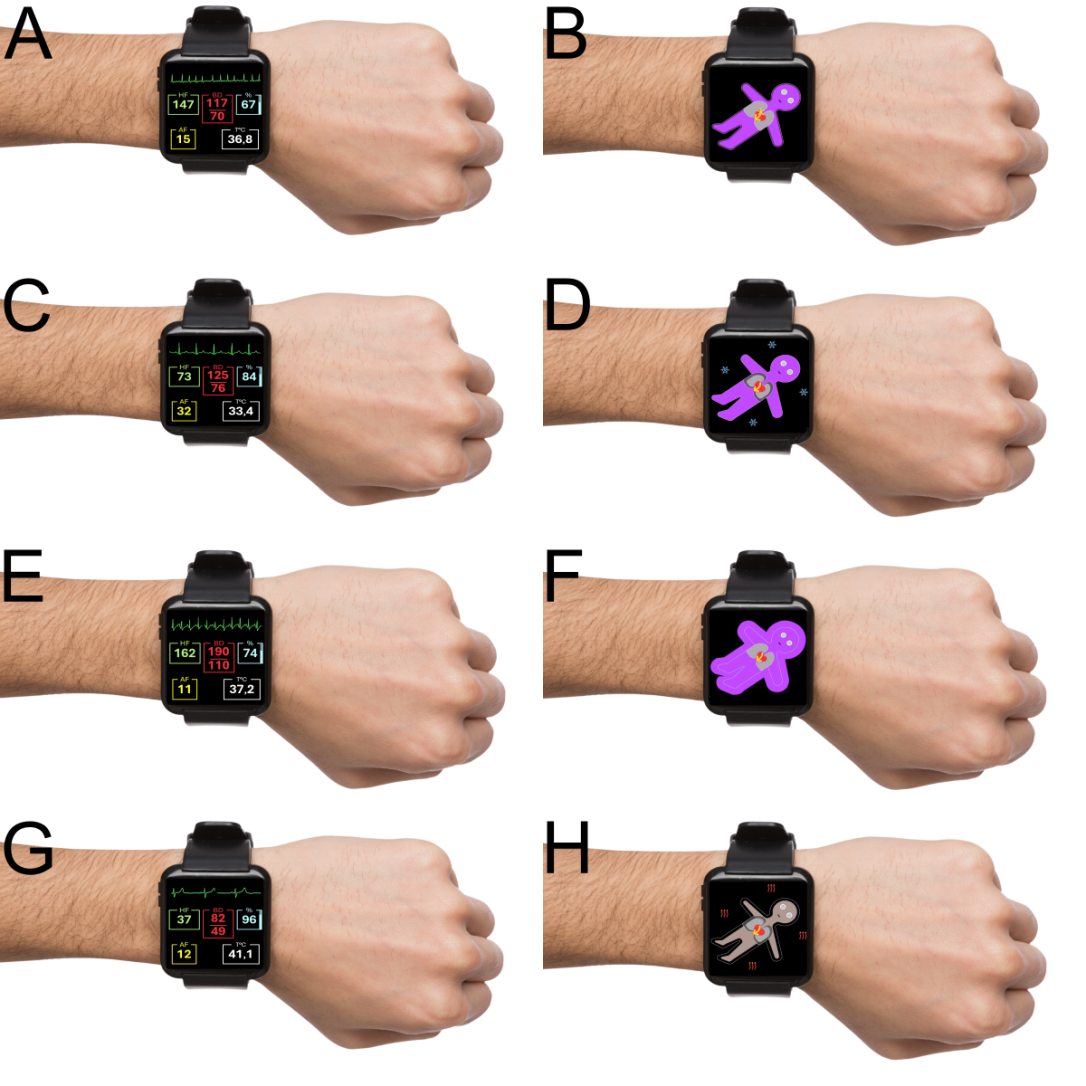
Visualizations for conventional monitoring (A, C, E, and G) and the Visual Patient Wearable system (B, D, F, and H), showcasing the same pathologies (scenarios 1 to 4).

Figure 1 shows a side-by-side comparison of both digital visualizations. Figure S1 in Multimedia Appendix 1 shows the flowchart of the study design. Table S1 in Multimedia Appendix 1 shows the vital sign deviations and cutoff values.

Participants were approached directly on the ward and invited to participate, resulting in a convenience sample of readily available and willing patients. They first completed an assessment of general knowledge of vital signs, based on conventional monitoring values and alarm thresholds. Participants were then randomly assigned to either a Visual Patient Wearable group or a conventional monitoring group using a computer-generated random number sequence [26]. Before testing, each participant individually viewed a standardized instructional video corresponding to their assigned visualization method (refer to Multimedia Appendix 2 for the Visual Patient Wearable system and Multimedia Appendix 3 for conventional monitoring). Subsequently, participants were exposed to 4 standardized scenarios (Figure 1) presented in an individually randomized order, again determined using a computer-generated random number sequence from 1 to 4 [26]. Each scenario was displayed for 6 seconds to simulate a glance-based assessment (eg, following an alarm). After each display, participants were required to recall the status of each vital sign (too low, normal, or too high). Participants allocated to the Visual Patient Wearable group subsequently completed a questionnaire consisting of 6 statements addressing the design and applicability of the Visual Patient Wearable system (Textbox 1).

**Questionnaire assessing participants’ perceptions of the Visual Patient Wearable system.**

- Question 1: “I would consider the avatar a useful addition to a wearable device such as a smartwatch.”
- Question 2: “Using the avatar would improve my perception of the vital signs.”
- Question 3: “Using the patient avatar would improve my ability to manage my health.”
- Question 4: “The patient avatar was clear and understandable.”
- Question 5: “The patient avatar would be easy for me to use.”
- Question 6: “Learning to understand and use the patient avatar would be easy for me.”

These statements were rated on a 5-point Likert scale from 1 (strongly disagree) to 5 (completely agree).

Overall, 46% (31/67) of the participants underwent surgery. Among them, 23% (7/31) reported experiencing visual patient avatars during their procedure, accounting for 10% (7/67) of all participants. These participants subsequently completed 6 additional questions, rated on a 7-point Likert scale ranging from 1 (strongly disagree) to 7 (completely agree; Textbox 2).

Finally, demographic data (sex, age, and study center) were collected.

**Additional questionnaire assessing participants’ perceptions of the visual patient avatar system.**

- Question 1: “The visual patient avatar confused me.”
- Question 2: “The visual patient avatar cheered me up.”
- Question 3: “The visual patient avatar scared me.”
- Question 4: “The visual patient avatar seemed unprofessional.”
- Question 5: “The visual patient avatar made me feel well-monitored.”
- Question 6: “The visual patient avatar gave me a positive impression.”

### Data Collection

The study assessed the suitability of the experimental technology solely for its intended patient user group. Participants’ medical history, current illness, or treatment data were neither collected, stored, nor influenced. Data were collected digitally using offline-capable software (Harvest Your Data) together with a single instructor. Testing lasted approximately 30 minutes per participant. No data could be traced back to individual participants. The following data were recorded: recall of vital signs; pretesting knowledge of conventional monitoring aspects (physiological thresholds of vital signs); answers from Likert rating scales; and demographics, including age (in years), sex (male or female), chronic illness (yes or no), surgery (yes or no), and experience with visual patient avatars in the operating room (yes or no).

### Study Centers

This study was conducted at 3 academic medical centers: the University Hospital Zurich, Switzerland; the University Hospital Bonn, Germany; and the University Hospital Frankfurt am Main, Germany.

### Study Participants

Patients were approached on general wards at participating centers and informed about the study. All participants received written information about the study procedures and data handling and provided written informed consent before participating. We defined the following inclusion criteria: adults (≥18years) hospitalized on a general ward, fluent in German, and fully conscious (Glasgow Coma Scale=15). Exclusion criteria were as follows: admission to intermediate or intensive care units, any cognitive impairments (eg, dementia, intellectual disability, delirium, or language barrier), thought disorders, inability or unwillingness to provide informed consent, uncorrected visual impairment (unable to read a newspaper), or acute psychological or physical distress.

### Statistical Analysis

For the a priori sample size calculation, we assumed a clinically relevant difference of 2 correctly identified vital signs between groups. On the basis of this assumption, with a power of 80% and a significance level of *P<*.05, 31 participants per group (62 in total) would be required. This estimate was based on the expected performance with the conventional monitoring device and the anticipated improvement using the Visual Patient Wearable system. All analyses were performed at the participant level. For each participant, responses were first aggregated across all scenarios to yield a single summary value per vital sign as well as 1 overall correctness value, ensuring that each participant contributed only 1 observation to the final analyses. Descriptive statistics were reported as medians with IQRs (Q1-Q3) for continuous variables and as counts and percentages for categorical variables. The primary outcome was the number of correctly identified vital sign deviations, reported both per signal (respiratory rate, SpO_2_, temperature, blood pressure, heart rate, and heart rhythm) and overall. Comparisons between the 2 modalities (Visual Patient Wearable vs conventional monitoring) were performed using the Wilcoxon rank sum test for independent samples (MATLAB function *ranksum*), applied separately for each vital sign and for the overall mean score. To quantify relative performance, risk ratios with 95% CIs (2.5th and 97.5th percentiles) were calculated using nonparametric bootstrapping (10,000 iterations). The risk ratio was defined as the proportion of correct identifications in the Visual Patient Wearable condition relative to the conventional condition, calculated both per signal and overall. To evaluate potential influences of participant demographics or study center, we conducted nonparametric analyses. Center effects were assessed using the Kruskal-Wallis test on overall correctness. Age effects were evaluated using Spearman correlation, and sex differences were assessed with the Wilcoxon rank sum test. To confirm the robustness of the primary Visual Patient Wearable vs the conventional effect, sensitivity analyses were performed, stratified by age (median split) and center, comparing each stratum using Wilcoxon rank sum tests. In addition, to assess potential familiarity effects, overall correctness was compared between participants with and without previous exposure using Wilcoxon rank sum tests, analyzed separately for each study arm. All statistical analyses and visualizations were performed in MATLAB R2023a (update 8, MathWorks).

### Creation of Visual Patient Wearable System

The Visual Patient Wearable system uses an animated patient-avatar display based on existing visual patient avatars [27], which represent numerical vital signs in an animated format. Only minor modifications, regarding the size of thoracic organs, were made for the Visual Patient Wearable system to better fit the smaller screen. It visualized 6 vital signs: respiratory rate, SpO_2_, temperature, blood pressure, heart rate, and heart rhythm, which most wearable devices can measure. Respiratory rate was indicated by the speed of lung movements (slow=low and fast=high). Oxygen saturation was represented by skin color (brown ≥92% and purple or cyanotic <92%). Temperature was indicated by the presence of ice crystals or heat waves surrounding the avatar; the absence of these showed a normal temperature. Blood pressure was conveyed by the overall filling of the avatar (thin=low and thick=high). Heart rate was displayed via the speed of electrical excitation, visualized as a yellow arrow in the heart. Heart rhythm was represented by the excitation pattern: regular propagation from atrium to ventricle indicated sinus rhythm, whereas irregular atrial discharges, shown as lightning flashes near the sinus node, indicated atrial fibrillation.

Animated examples of the Visual Patient Wearable system are provided in Multimedia Appendix 2, and cutoff values for low, normal, and high readings are listed in Table S1 in Multimedia Appendix 1.

For comparison, the conventional numerical display presented the same 6 vital signs in standard formats, with values, waveforms (electrocardiogram), and colors, reflecting normal or abnormal ranges. The corresponding conventional display of the 6 vital signs is detailed in Multimedia Appendix 3. The Visual Patient Wearable system’s display and conventional displays conveyed equivalent information content, allowing observed differences in user performance to be attributed to the mode of visualization rather than differences in data availability or labeling.

## Results

### Overview

A total of 67 patients were recruited into the study. There were no dropouts, and all data (1584 responses, 792 per arm) were included in the analysis. Demographic and baseline characteristics of the participants are presented in Table 1. The median overall percentage of correct responses was 66.7% (IQR 49%-79.2%) in the Visual Patient Wearable group and 45.8% (IQR 33.3%-62.5%; *P*<.001) in the conventional group. Baseline knowledge of physiological threshold values for vital signs (heart rate, respiratory rate, systolic and diastolic blood pressure, SpO_2_, and body temperature) was moderate across the study population, and there were no statistically significant differences between groups (Figure S2 in Multimedia Appendix 1; *P*=.74). The age of participants was not significantly different between the Visual Patient Wearable and conventional monitoring groups (Visual Patient Wearable group: median age 36 [IQR 23-48] years; conventional group: median age 45 [IQR 30-55] years; *P*=.15). Correctness differed significantly across centers (Kruskal-Wallis; *P*<.001), indicating some heterogeneity in performance. Nevertheless, within each center, the Visual Patient Wearable group still consistently achieved significantly higher correctness than the conventional group (Table S2 in Multimedia Appendix 1). Age was also significantly associated with correctness, exhibiting a moderate negative correlation (Spearman rho=–0.374; *P*<.001). However, in age-stratified analyses, the Visual Patient Wearable group outperformed the conventional group in both younger participants (conventional group: median 50%, IQR 50%-67% vs Visual Patient Wearable group: median 67%, IQR 50%-83%; *P*<.001) and in older participants (conventional group: median 33%, IQR 17%-50% vs Visual Patient Wearable group: median 50%, IQR 33%-67%; *P*=.015). No significant differences in performance were observed between female and male participants, independent of center (*P*=.70). Within the Visual Patient Wearable group, participants with previous Visual patient avatar experience (13/67, 19%) achieved higher overall correctness than those without experience (54/67, 81%; median 83.3%, IQR 67%-92% vs median 66.7%, IQR 50%-83%; *P*=.02), indicating a benefit of familiarity. In contrast, in the conventional group, performance was similar regardless of previous exposure (median 50%, IQR 50%-67% vs median 50%, IQR 42%-67%; *P*=.80).

**Table 1:** Study participants’ demographics (N=67).

Parameter		Visual Patient Wearable group (n=34)	Conventional group (n=33)
**Location, n (%)**			
	University Hospital Bonn	10 (29)	10 (30)
	University Hospital Frankfurt	11 (32)	10 (30)
	University Hospital Zurich	13 (38)	13 (39)
Age (years), median (IQR)		36 (23-48)	45 (30-55)
Female sex, n (%)		20 (59)	12 (36)
Prior surgery at the center, n (%)		17 (50)	15 (45)
Any chronic illness, n (%)		9 (26)	12 (36)
Previous visual patient avatar exposure, n (%)		7 (21)	6 (18)

### Primary Outcome: Correct Identification

The Visual Patient Wearable system improved correct identification across all 4 scenarios:

1. Conventional group (median 50%, IQR 16.7%-54.2%) vs Visual Patient Wearable group (median 66.7%, IQR 50%-83.3%; *P*=.004)
2. Conventional group (median 50%, IQR 33.3%-66.7%) vs Visual Patient Wearable group (median 66.7%, 50%-83.3%; *P*=.041)
3. Conventional group (median 50%, IQR 29.2%-66.7%) vs Visual Patient Wearable group (median 66.7%, IQR 50%-83.3%; *P*=.005)
4. Conventional group (median 50%, IQR 33.3%-70.8%) vs Visual Patient Wearable group (median 66.7%, IQR 50%-83.3%; *P*=.032; Figure S3 in Multimedia Appendix 1)

**Figure 2:**
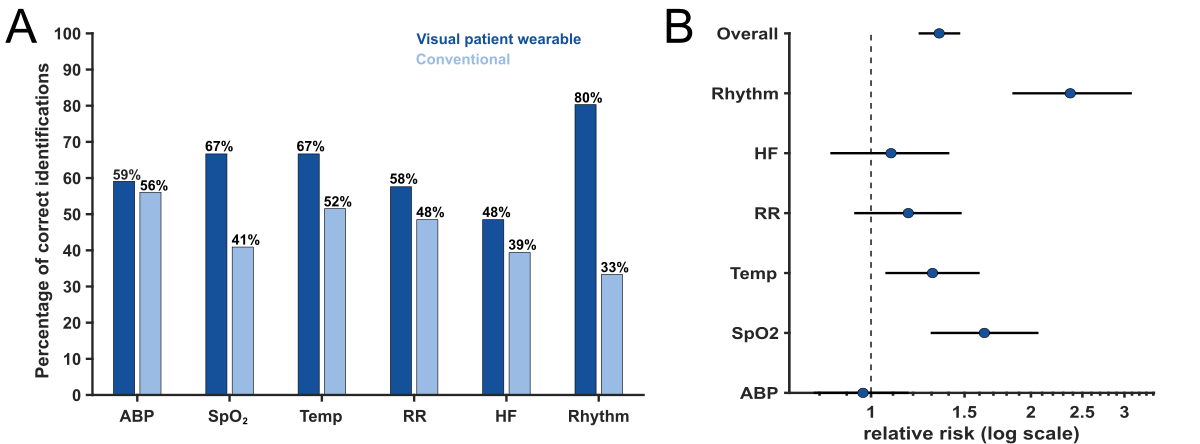
Primary outcome (correctness) comparing the Visual Patient Wearable system and conventional monitoring. (A) Bar chart displaying the percentage of correct identifications of vital sign deviations across all 4 scenarios for 6 parameters. (B) Log-scaled risk ratios per vital sign. The risk ratios were considered significant when the 95% CI did not include 1. ABP: arterial blood pressure; SpO2: oxygen saturation; Temp: temperature; RR: respiratory rate; HR: heart rate.

This was reflected in the overall increase in correct responses from a median of 45.8% (IQR 33%-63%) with the conventional group to a median of 66.7% (IQR 49%-79%) with the Visual Patient Wearable group (*P*<.001). Relative risk estimates indicated an overall 34% (95% CI 23%-47%) higher probability of a correct response when the Visual Patient Wearable system was used. The magnitude of this effect varied across signals, with the most considerable improvement observed for heart rhythm (137%, 95% CI 85%-209%), followed by SpO_2_ (64%, 95% CI 30%-108%) and temperature (30%, 95% CI 5%-60%). Heart rate, respiratory rate, and blood pressure showed no significant change in risk ratios. For a complete statistical analysis of all vital sign parameters, refer to Figure 2 and Table 2.

**Table 2:** Statistical overview of percentages of correctly identified vital signs over 4 scenarios (N=67).

Signal	Visual Patient Wearable (%), median (IQR)	Conventional (%), median (IQR)	Risk ratio (95% CI)	Benefit of the Visual Patient Wearable system
Arterial blood pressure	50 (50-75)	50 (50-75)	0.97 (0.79-1.18)	None
Oxygen saturation	75 (44-100)	25 (25-75)	1.64 (1.30-2.08)	Moderate
Temperature	75 (25-100)	50 (25-50)	1.30 (1.05-1.60)	Moderate
Respiratory rate	50 (50-75)	50 (50-75)	1.18 (0.93-1.48)	None
Heart rate	50 (25-75)	50 (25-75)	1.09 (0.84-1.40)	None
Rhythm	75 (70-100)	25 (25-50)	2.37 (1.85-3.09)	Strong
Overall	66.7 (49-79)	45.8 (33-63)	1.34 (1.23-1.47)	Moderate

### User Experience and Feedback

We conducted 2 separate evaluations of the Visual Patient Wearable system. The first focused on general user impressions and was based on a 5-point Likert scale. Participants rated various aspects of the system as follows: visualization had a median score of 4 (IQR 4-5), organization had a median score of 4 (IQR 3-5), usefulness had a median score of 4 (IQR 4-5), learnability had a median score of 4 (IQR 4-5), understandability had a median score of 4 (IQR 3.75-5), and usability had a median score of 4.18 (IQR 4-5; Figure S4 in Multimedia Appendix 1). These results indicate that all evaluated aspects were judged positively, with particularly strong ratings for learnability, usability, and visualization.

In a separate analysis, we evaluated feedback from participants who had personally experienced visual patient avatars in the operating room, using a 7-point Likert scale (with higher scores indicating more favorable ratings, except for negative aspects such as fear, confusion, and untrustworthy appearance). These participants reported an overall positive impression, with a median score of 5 (IQR 4.5-6), and felt well monitored, with a median score of 5 (IQR 4.75-6). The visual patient avatars were also perceived as somewhat cheerful, with a median of 5 (IQR 4-6.25). The ratings for negative aspects were consistently low: fear was rated at 1 (IQR 1-2), confusion at 2 (IQR 1-2.5), and untrustworthy appearance at 2 (IQR 1-2.5). Participants who encountered visual patient avatars in clinical practice experienced them positively, with minimal negative perceptions (Figure S5 in Multimedia Appendix 1).

## Discussion

### Principal Findings

In this simulation study conducted on a general ward patient cohort, the Visual Patient Wearable system was associated with higher accuracy in detecting vital sign deviations compared to conventional monitoring visualizations. The greatest improvements were seen for heart rhythm, followed by SpO_2_ and temperature. Heart rate, respiratory rate, and blood pressure showed nonsignificant variability in favor of better detection with the Visual Patient Wearable system. Qualitative assessments indicated that patients perceived the Visual Patient Wearable system as understandable and easy to learn and believed it had potential value for future real-world clinical use.

Our primary outcome was the detection and recall of changes in simulated vital signs, averaging across all 6 parameters, to assess the overall effect of the avatar. The effect varied across parameters for 2 main reasons. First, some vital signs were more visually prominent on the wearable device. For example, SpO_2_ changes were clearly indicated by a color shift in the avatar’s skin, making them noticeable even on a small display. In contrast, parameters such as respiratory rate and heart rate were represented only on the avatar’s thorax, appearing smaller and less visually prominent when shown on a small screen. Second, participants’ general knowledge of common vital signs may have influenced detection. Blood pressure was generally well recognized, whereas electrocardiogram signals and heart rhythm, typically reserved for medical professionals, were difficult to interpret with conventional displays. However, the simplified Visual Patient Wearable visualization allowed participants to correctly identify the heart rhythm much more reliably.

Continuous vital sign monitoring has gained increasing attention for its potential to improve patient safety and clinical outcomes, particularly in anesthesiology, where perioperative care is crucial for preventing morbidity and mortality [28-30]. Continuous monitoring has been shown to detect critical events more quickly and reduce adverse events, such as clinical deterioration and cardiac arrests, compared to intermittent monitoring [31,32]. Advances in sensor technology [22] and machine learning [33] further highlight the growing interest in continuous ward monitoring. Unlike sensor- or algorithm-specific solutions, our Visual Patient Wearable visualization prioritizes patient comprehension, offering an intuitive and co-designed interface with the goal of enhancing usability, relevance, and acceptance [34]. The Visual Patient Wearable system aims to empower patients to actively engage in monitoring their health, supporting early detection of deviations and potentially mitigating risks associated with delayed intervention [35]. This study specifically focused on the first link in the intervention-outcome chain: patient detection of vital sign deviations. Subsequent steps, such as alerting clinicians, process measures such as response time or escalation, and ultimate clinical outcomes, were not assessed and will be addressed in future clinical trials to evaluate the full impact of the Visual Patient Wearable system on clinical workflows and patient care, as this study had no influence on real clinical behavior or outcomes. The next step will be to conduct a feasibility study on ward patients using a Visual Patient Wearable prototype, explicitly considering real-world conditions such as variations in lighting, background noise, alarms, and typical workflow interruptions. Practical end points will include patient adherence, time-to-notice of vital sign deviations, usability, and satisfaction. The study will also explore how the Visual Patient Wearable system can be integrated into standard patient monitoring routines, notifying staff when necessary, without disrupting care, and providing critical insights into its feasibility and potential application in clinical practice. In addition, integrating machine learning algorithms and optimizing data flow to the responsible health care professional into the Visual Patient Wearable system could further enhance its predictive capabilities [36,37].

### Situational Awareness and Visual Patient Avatar Design

Visualization plays a crucial role in presenting complex data in an intuitive manner, thereby reducing cognitive load for clinicians and patients. The Philips visual patient avatar integrates multiple vital signs into a single animated figure [38]. Simulation studies indicate that user-centered displays, using color, shape, and motion, assist anesthetists in swiftly identifying clinical deteriorations, increasing diagnostic confidence, and reducing workload [1,8,9]. These visuals are designed to leverage innate human abilities in color and motion recognition, offering a rapid overview of multiple vital sign data [39].

While visual patient avatars were initially developed for clinical professionals, the same design principles may also apply to patient-facing systems. In particular, presenting physiological information in a simplified and intuitive visual form may help patients better understand their own health data. Improved comprehension of physiological information through accessible visualizations may increase engagement with health-related information and self-care, with potential benefits for both mental and physical health. Support for this concept can be found in previous work on adaptive digital health interventions. For example, a psychoeducational mobile health program for parents expecting a fetus classified as small for gestational age demonstrated that providing tailored, understandable health information during pregnancy was associated not only with improved emotional support for the mother but also with higher birth weights in the intervention group compared with controls [40]. This suggests that improved comprehension and engagement with health information may have downstream effects beyond psychological well-being.

### Limitations

This study has several limitations. The sample size was limited, and the cohort was relatively young. Participants were recruited as a convenience sample, which inherently restricts the generalizability of the findings. While inclusion of participants from 3 academic centers in different countries increased heterogeneity, performance differences were observed between centers and age groups, underscoring the potential influence of local training environments and demographic factors. Group allocation and scenario order were randomized, and no significant differences in age or previous knowledge of vital sign thresholds were present between groups. Using short, 6-second video scenarios to simulate clinical scenarios may not fully reflect the complexity of real-world decision-making for patients to alert health care providers. A future clinical study using a functional prototype is warranted. Previous exposure to the Visual Patient Wearable system was limited to a short instructional video, and participants may not have reached full proficiency before testing. Thus, their performance may not reflect actual use after more extended training or repeated use. In addition, we were unable to assess participants’ long-term learning or retention, as repeated sessions and follow-up assessments were not feasible in this study. Consequently, the results may not capture potential improvements in detection accuracy or usability that could occur with extended exposure and repeated use over time. The evaluation of the Visual Patient Wearable system included self-reported ratings based on hypothetical use scenarios, which may not accurately predict behavior or acceptance in real clinical environments. The subset of participants who experienced visual patient avatars as surgical patients was small (approximately 7/67, 10%), and retrospective recall of emotional reactions may be prone to bias. There were challenges related to the Visual Patient Wearable system, such as data security concerns, patient acceptance, and usability issues among older or technologically inexperienced populations [41]. Addressing these challenges is crucial for ensuring the widespread adoption and effective implementation of the Visual Patient Wearable system in routine clinical practice.

### Conclusions

This computer-based study evaluated Visual Patient Wearable visualizations for continuous monitoring of vital signs in patients. Our findings indicate that the Visual Patient Wearable system may improve the recognition of simulated vital sign deviations, even in complex scenarios. Participants generally found the system intuitive, learnable, and reassuring, with minimal negative emotional responses. These results suggest that the Visual Patient Wearable system could help patients better understand their vital sign data and communicate relevant information to staff, although our study was not based on real clinical outcomes. Conventional monitoring systems are primarily designed for health care professionals and often present information that is not easily comprehensible to patients. The Visual Patient Wearable system addresses this gap by providing an intuitive, at a glance interface that enables patients to engage with their monitoring. The next step should be to refine the visualization for small screens, develop a software prototype compatible with common wearable devices, and evaluate its impact in a clinical study using patients’ data.

## Appendix

Multimedia Appendix 1 Study flowchart and additional figures illustrating the study outcomes.

Multimedia Appendix 2 Standardized training video for the Visual Patient Wearable.

Multimedia Appendix 3 Standardized training video for conventional monitoring.

## Abbreviations

CONSORT: Consolidated Standards of Reporting Trials
SpO2: oxygen saturation
STROBE: Strengthening the Reporting of Observational Studies in Epidemiology

## Data Availability

The datasets generated or analyzed during this study are available from the corresponding author on reasonable request.
