# Supplementary material for: Using a Wearable-Based Animated Patient Avatar to Improve Patients’ Perception of Vital Signs: Multicenter Computer-Based Study
Source: JMIR Form Res. 2026 Mar 12;10:e84130. doi: 10.2196/84130 (PMC13022546; doi:10.2196/84130)
Supplement: Multimedia Appendix 1 [file formative_v10i1e84130_app1.docx]

**Multimedia Appendix 1:****Using a wearable-based animated patient avatar to improve patients’ perception of vital sign information.**

# Johannes Köhler^†^, Max Ebensperger^†^, Cynthia A. Hunn, Achilles Delis, Florian Piekarski, Florian J. Raimann, Gregor Massoth, Kai Zacharowski, David W. Tscholl, Tadzio R. Roche^*^

† These authors contributed equally to this publication

***Corresponding author:**

Tadzio R. Roche

Email: tadzio.roche@gmx.de

Running title: Visual Patient Avatar Improves Vital Sign Awareness in Patients

**Table S1: Scenarios and vital sign deviations** Abbreviations: ABP, Ambulatory Blood Pressure; RR, Respiratory Rate; HF, Heart Rate.

| **Scenario** | **RR** | **HR** | **SpO2** | **ABP** | **Rhythm** | **Temperature** |
| --- | --- | --- | --- | --- | --- | --- |
| A, B | normal | high | low | normal | AFib | normal |
| C, D | high | normal | low | normal | normal | low |
| E, F | normal | high | low | High | Tachycardia | normal |
| G, H | normal | low | normal | low | Bradycardia | high |

**Table S2: Results of correctness for Conventional and Visual Patient Wearable per center.**

|  | Conventional correct median percentage [Q1, Q3] | VPW correct median percentage [Q1, Q3] | P-value |
| --- | --- | --- | --- |
| Center 1 | 50% [42, 67] | 67% [50, 83] | <0.001 |
| Center 2 | 50% [50, 75] | 75% [50, 83] | 0.007 |
| Center 3 | 25% [17, 42] | 33% [33, 50] | 0.004 |

**
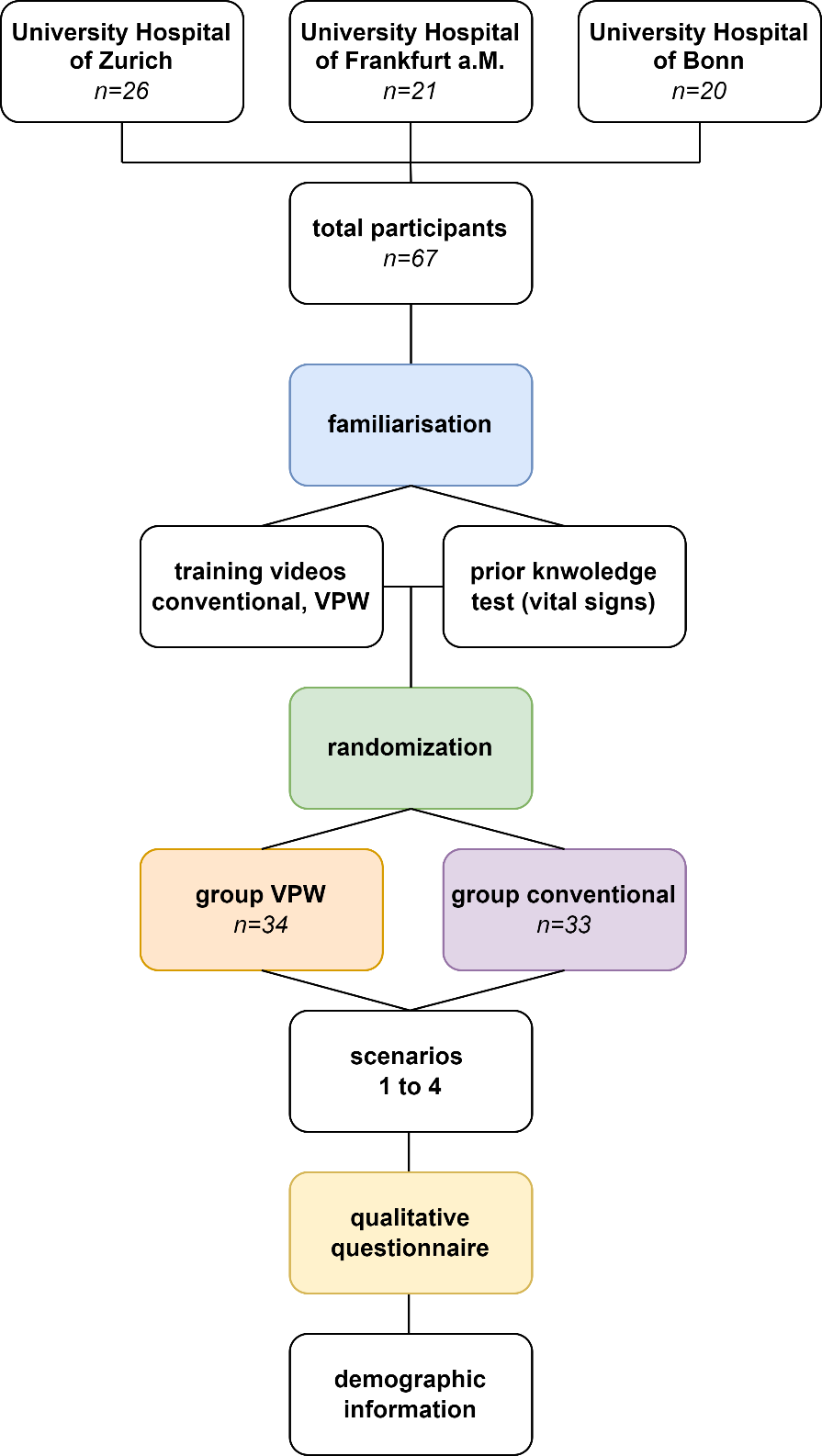

Figure S1: Flowchart of Study design**

**
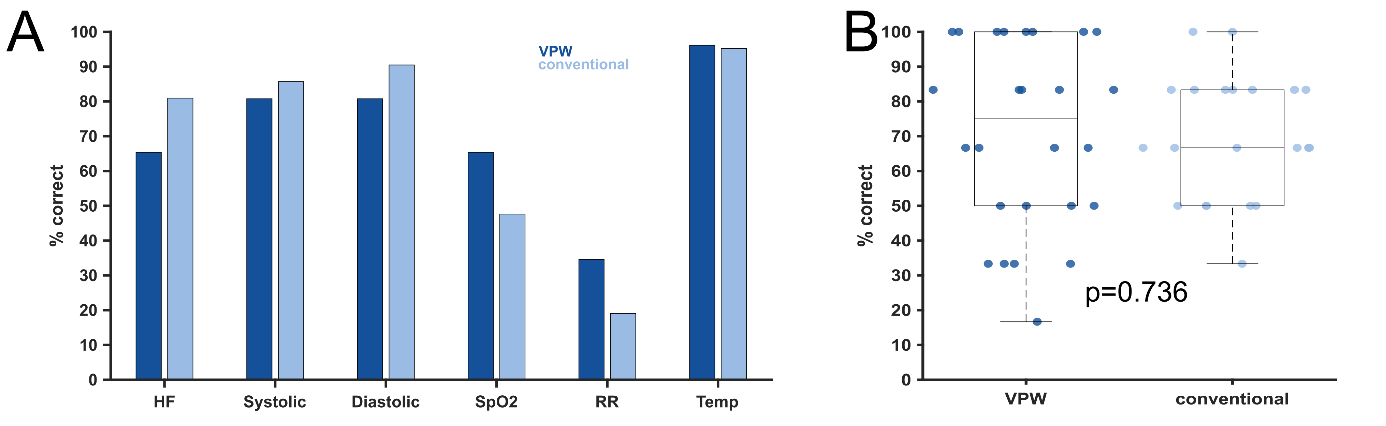
**

**Figure S2: Bar Chart and Boxplot measuring prior knowledge of participants of physiological (“*normal*”) ranges of different vital signs.**

**A)** Bar charts for the different vital signs

**B)** Box charts indicating no significant difference for prior knowledge (p=0.736).


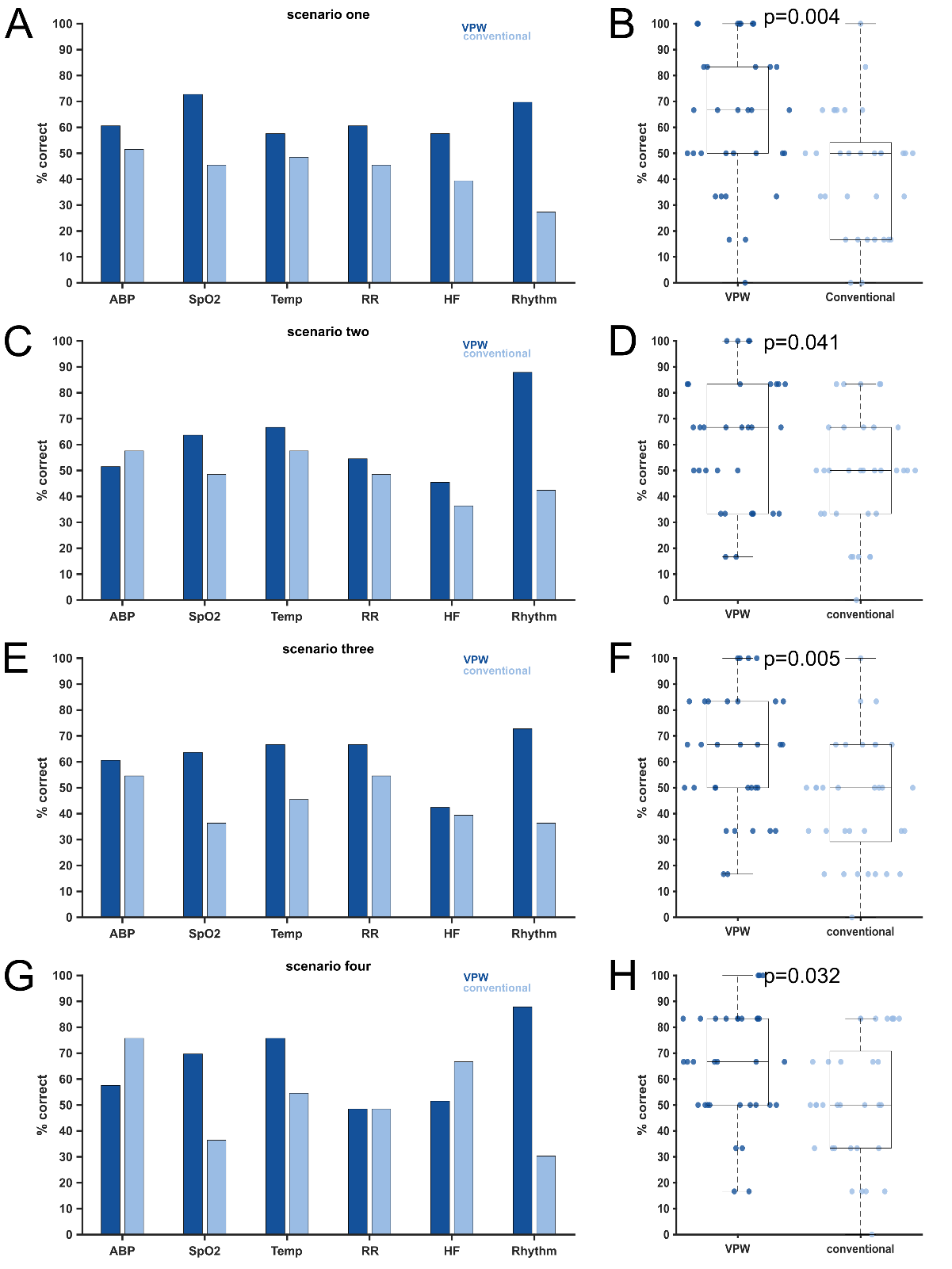


**Figure S3: Bar charts and Boxplots indicating percentage correctness for different scenarios**

**A)** and **B)** Scenario 1

**C)** and **D)** Scenario 2

**E)** and **F)** Scenario 3

**G)** and **H**) Scenario 4

**
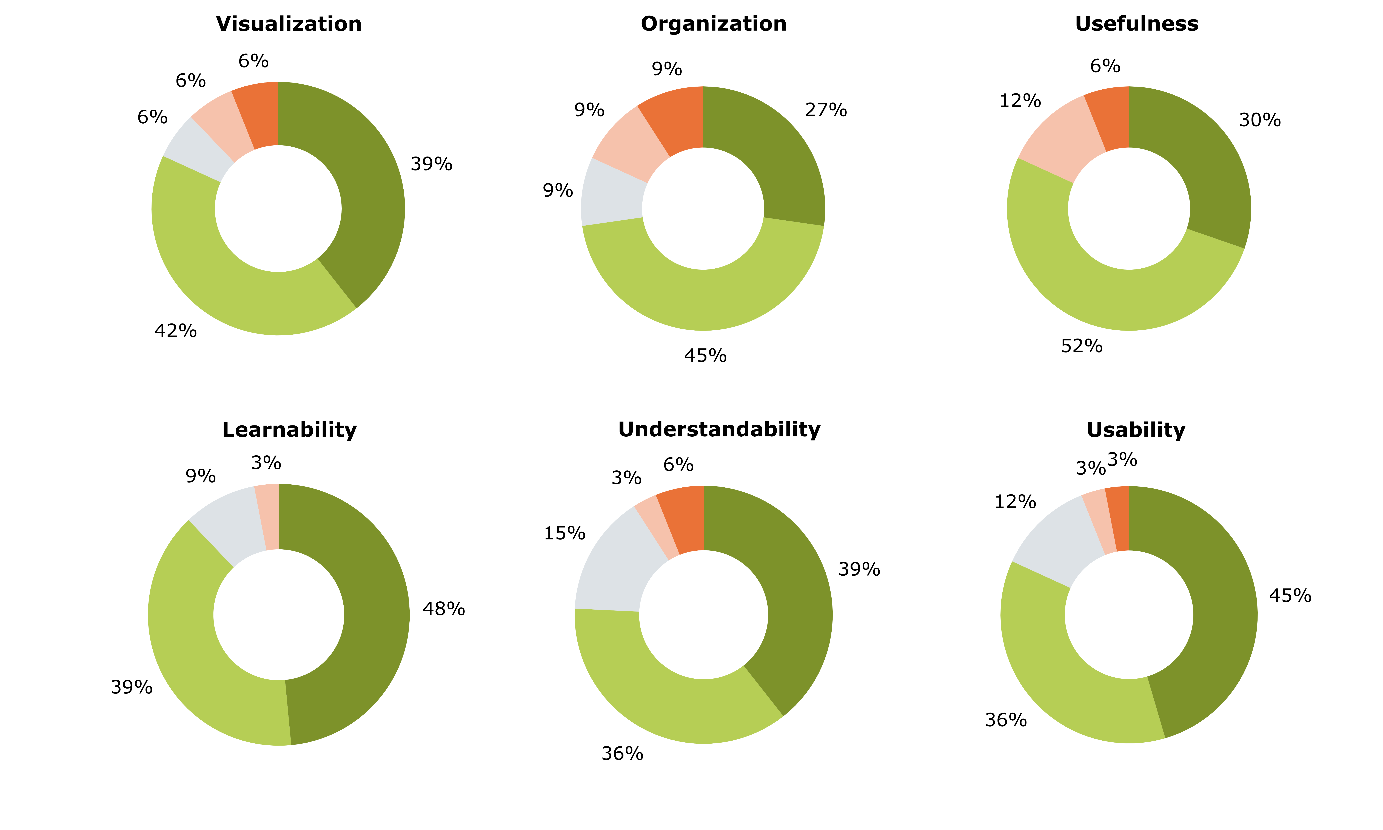
**

**Figure S4: Donut charts showing Likert rating scale results (maximum 5 points) for different aspects of the Visual Patient Wearable.** Colors: dark green (5 points), light green (4 points), gray (3 points), light red (2 points), and dark red (1 point).


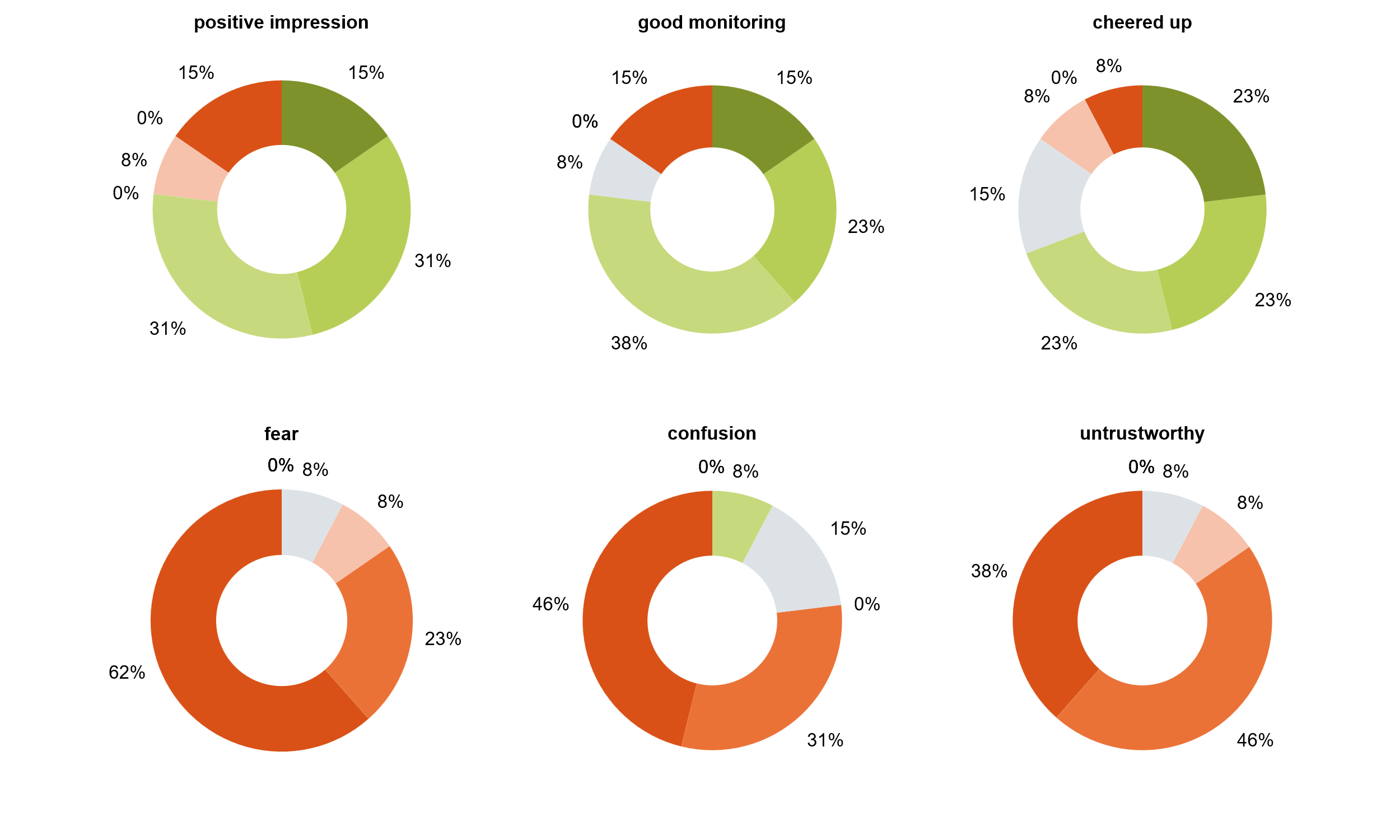


**Figure S5: Donut charts illustrating Likert scale ratings (maximum score: 7) for various aspects of the Visual Patient Avatar, based on participants’ firsthand experiences during their own anaesthesia in the operating room.** Colours: dark green (7 points), green (6 points), light green (4 points), grey (3 points), red (2 points), light red (1 point), no colour (0 points, 0% for all categories).
